# High-purity lignin isolated from poplar wood meal through dissolving treatment with deep eutectic solvents

**DOI:** 10.1098/rsos.181757

**Published:** 2019-01-23

**Authors:** Yujie Chen, Lili Zhang, Juan Yu, Yingzhao Lu, Bo Jiang, Yimin Fan, Zhiguo Wang

**Affiliations:** 1 Jiangsu Co-Innovation Center of Efficient Processing and Utilization of Forest Resources, Nanjing Forestry University, Nanjing 210037, People's Republic of China; 2 College of Light Industry and Food Engineering, Nanjing Forestry University, Longpan Road 159, Nanjing, People's Republic of China

**Keywords:** deep eutectic solvents, lignin isolation, selective extraction, high purity

## Abstract

Deep eutectic solvents (DESs) have potential applications in biomass conversion and green chemicals due to their cost-effectiveness and environmentally friendly properties. This study reports on a feasible method of using DESs for lignin selective extraction from poplar wood meal. DESs obtained from various hydrogen-bond donors and acceptors were used to evaluate the dissolving capacity of lignin from poplar wood meal. Among the various DESs, lactic acid: choline chloride (9 : 1) exhibits the optimal extraction capacity, which is capable of selectively dissolving 95% of lignin from poplar wood meal at 120°C for 6 h. The purity of isolated lignin reaches 98% after regeneration in water. From Fourier Transform-IR, nitrobenzene oxidation and nuclear magnetic resonance analysis, the results demonstrate that the DESs can selectively cleave ether linkages and damage the non-condensation section of lignin, thereby facilitating lignin dissolution from wood meal. Thus, this study provides a promising route for the extraction of high-purity lignin from biomass materials.

## 1. Introduction

Lignocellulose, as the most abundant renewable resource on Earth, has received considerable attention due to its high-value product conversion [1]. Developing sustainable ‘green’ technology to use lignocellulose has become an important concern at present. However, lignocellulose, which is composed of lignin, hemicellulose and cellulose, is considered to possess a complex physical structure in which lignin can bind the cellulose and hemicellulose together to form a cross-linked polymer network. Moreover, numerous chemical and hydrogen bonds exist between lignin and these carbohydrates. The structural complexity and inhomogeneity of lignocellulose strongly limits the effective separation and utilization of the lignocellulose components [2]. Therefore, pursuing a suitable technology for separating lignocellulose components is a significant prerequisite for the conversion of lignocellulose into valuable materials.

A deep eutectic solvent (DES) is a homogeneous mixture made by two solid chemicals in which the melting point of the final mixture is lower than those of the individual compounds [3]. A DES is a type of new solvent system with low volatility, non-toxicity and low cost, which is composed of a hydrogen-bond acceptor (HBA) and a hydrogen-bond donor (HBD). Abbott *et al*. prepared eutectic mixtures of salts by mixing carboxylic acids and choline chloride (ChCl), which can retain their liquid states at the ambient temperature [4]. The physical performances of the DES mixture, such as its surface tension, conductivity and viscosity, were studied, thereby demonstrating that the reduction of the melting point is attributed to the formation of hydrogen bonds between two compounds. Compared with conventional ionic liquids, the DESs possess various favourable properties (ease of synthesis, biodegradability and availability) and thus are capable of becoming versatile alternatives to ionic liquids.

The most recent discovery of the DES is that it can be applied to lignin extraction [5]. Few studies suggest that the DES is capable of dissolving lignin from woody plants by breaking most β-O-4 bonds between the lignin and carbohydrate, thereby realizing the separation of lignocellulose components [6,7]. Moreover, due to the interaction principles of HBA and HBD, the ether linkages among phenyl propane units are readily cleaved to generate low-molecular-weight lignin, which may be due to the acid catalysis behaviour of HBD [5]. In other words, DESs can serve as a promising alternative solvent for the separation of lignocellulose and high-value lignin production. Francisco *et al*. used as-prepared lactic acid–choline chloride mixtures to dissolve multiple lignocelluloses, thereby suggesting that DES has high selectivity for the separation of lignin and cellulose [6]. Alvarez-Vasco *et al*. prepared a DES by mixing ChCl and four HBDs (glycerol, levulinic acid, lactic acid and acetic acid) for treating hardwood and softwood. The results suggested that these DESs are preferred to extract lignin from plants and the obtained lignin exhibited unique structural properties with 95% purity [5]. Yiin *et al*. studied the effects of water on the physiochemical properties of DESs, thereby demonstrating that the presence of little water enhances the lignin solubility of DES and has no effect on its thermal stability [8].

This work provided a feasible method of using DESs for lignin selective extraction from wood meal. Various DESs were prepared to dissolve poplar wood meal, and the lignin solubility of these DESs were compared to find the most effective DES for selective lignin dissolution. The effects of the treatment temperature and time on the extraction capacity of lignin were investigated to determine the optimal dissolving conditions. In addition, the structure of the extracted lignin was characterized by Fourier Transform-IR (FT-IR), nitrobenzene oxidation and nuclear magnetic resonance (NMR) analysis to verify the possible dissolving mechanism of lignin in the selected DES.

## 2. Material and methods

### 2.1. Materials

Poplar wood meals (40–80 mesh) were subjected to the extraction of benzene and ethanol for removing the extractive in wood meal prior to DES treatment (GB/T2677.6). From the component analysis, poplar wood meal was detected to contain 27.69% lignin, 41.22% cellulose and 20.47% hemicellulose [9]. Choline chloride (ChCl), DL-lactic acid (LA), urea, glycerol, citric acid monohydrate, glycol, succinic acid and acetamide were purchased from Shanghai Aladdin Chemical Co., Ltd., China. All other reagents were of analytical grade and were used without further purification.

### 2.2. Synthesis of DESs

Various hydrogen-bond donors and the hydrogen-bond acceptors based on the specific mole ratio were mixed and placed in a conical flask with magnetic stirring at 80°C for a definite time. All DESs mixtures became clear and were later stored at room temperature.

### 2.3. Solubility of wood meal in various DESs

Poplar wood meal (2 g, oven dry) was added into the conical flask with different DESs (98 g) and was stirred continuously at 120°C for 6 h. After dissolving, the solution and solid residue were separated by centrifugation at 5000 r min^−1^ for 10 min. The solid residue was washed using water and freeze-dried to evaluate the solubility of each DES. The solubility of DESs for poplar wood meal was called the dissolving removal rate (*S*)

2.1 $$S=\left(1-\frac{m_{1}}{M}\right)\times 100\text{\%},$$

where *m*_1_ is the mass of solid residue, and *M* is the mass of raw material.

### 2.4. Isolation of lignin with a lactic acid/choline chloride DES

**Figure 1.**
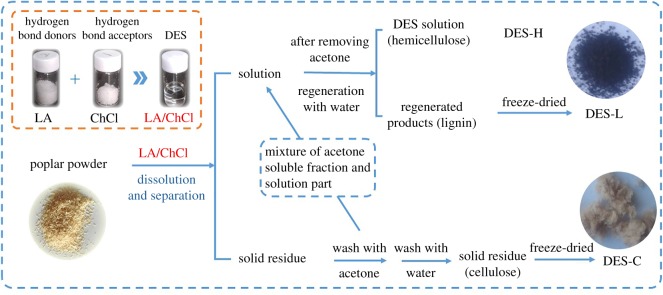
Procedure of lignin isolation from poplar with DES.

The process of lignin isolation using a LA/ChCl DES is shown in figure 1. The wood meal (2 g, oven dry) and LA/ChCl (98 g) were added into a conical flask with continuous stirring. The dissolving temperatures were 100°C, 110°C, 120°C and 130°C, and the dissolving times were 3 h and 6 h, respectively. After dissolving, the solution and solid residue can be separated by centrifugation. The solid residue was washed using acetone three times followed by washing using water and freeze-dried to obtain separated cellulose (DES-C, figure 1). The acetone soluble fraction was mixed with the solution part, and it removed acetone by rotary evaporation. The mixtures (acetone free) were regenerated by deionized water and freeze-dried to obtain the isolated lignin (DES-L, figure 1), while hemicellulose still remains in the solution (DES-H, figure 1).

The yield of solid residue (DES-C) and isolated lignin (DES-L) were calculated by the following equation:

2.2 $$F=\frac{m_{2}}{M}\times 100\text{\%},$$

where *m*_2_ is the mass of DES-C or DES-L, and *M* is the mass of raw material.

The solubility (S) of components in raw materials is determined based on the content of each component (cellulose, lignin and hemicellulose) in DES-C, as shown in the following equations:

2.3 $$\mathrm{The}\;\mathrm{solubility}\;\mathrm{of}\;\mathrm{cellulose}\;(\mathrm{SC}):\;\mathrm{SC}=\frac{\mathrm{OC}-\mathrm{RC}}{\mathrm{OC}}\times 100\text{\%},$$

2.4 $$\mathrm{The}\;\mathrm{solubility}\;\mathrm{of}\;\mathrm{lignin}\;(\mathrm{SL}):\;\mathrm{SL}=\frac{\mathrm{OL}-\mathrm{RL}}{\mathrm{OL}}\times 100\text{\%}$$

and

2.5 $$\mathrm{The}\;\mathrm{solubility}\;\mathrm{of}\;\mathrm{hemicellulose}\;(\mathrm{SH}):\;\mathrm{SH}=\frac{\mathrm{OH}-\mathrm{RH}}{\mathrm{OH}}\times 100\text{\%},$$

where OC, OL and OH are the respective masses of cellulose, lignin and hemicellulose in the original materials, and RC, RL and RH are the respective masses of cellulose, lignin and hemicellulose in solid residue (DES-C).

### 2.5. Lignin purity determination

Lignin is the total amount of acid soluble lignin (ASL) and acid insoluble lignin (AIL) in regenerated products (DES-L), according to standard methods [10]. The ASL was determined by UV-absorbance at 205 nm, which was performed on a UV–vis spectrophotometer (TU-1810, Puxi, Beijing, China). The content of sugar was measured by high-performance liquid chromatography (HPLC, Agilent 1200 Series, Santa Clara, CA). The lignin purity (PL) was calculated using the following:

2.6 $$\mathrm{PL}=\frac{\mathrm{RL}}{R}\times 100\text{\%},$$

where RL is the mass of lignin in regenerated products (ASL + AIL), and the R is the mass of regenerated products.

### 2.6. Characterization of isolated lignin (DES-L) and solid residue (DES-C)

The FT-IR spectra of DES-L and DES-C were obtained from the range of 4000–400 cm^−1^ on a VERTEX 80 V spectrometer (Bruker, Germany).

The nitrobenzene oxidation (NBO) of lignin was determined based on the method of Chen [11]. The milled wood lignin (MWL) was prepared as the control sample, which was obtained through the method of Björkman [12].

The structural characterization of purified DES-L and MWL by the ^13^C NMR was conducted on a BBO 600 MHz NMR spectrometer following standard procedures for lignin analysis [13]. The acetylated DES-L was analysed using deuterated dimethyl sulfoxide (DMSO-d6) as a solvent (200 mg sample per 0.5 ml DMSO-d6) with a small amount of relaxation agent (Cr(acac)_3_).

The ^1^H-^13^C two-dimensional NMRs (2D HSQC NMR) of the purified DES-L and MWL were performed on a Bruker 600 MHz instrument (AVANCE III, Switzerland) equipped with a cryogenically cooled 5 mm TCI z-gradient triple-resonance probe. The DES-L (50 mg) was dissolved into DMSO-d6 solvent (0.5 ml) according to the method of Kim *et al.* [14]. The spectral widths of ^1^H and ^13^C were from 0 to 16 ppm (9615 Hz) and from 0 to 165 ppm (24 900 Hz), respectively. The number of collected complex points was 2048 for the 1H-dimension with a recycle delay of 1.5 s. The number of transients was 64, and 256 time increments were recorded in the ^13^C-dimension. The chemical peak shift of DMSO (delta C 39.5 ppm, delta H 2.5 ppm) was used to calibrate the data. In this work, the relative proportions of the various joint bonds, methoxy and the S/G ratio of lignin were calculated using the aromatic ring as the internal standard.

The crystal structure of cellulose in DES-C was subjected to X-ray diffraction (XRD) in reflection mode with a diffraction angle of 2*θ* from 5° to 40° by a multifunction X-ray diffractometer (Ultima-IV) at 40 kV and 30 mA. The crystallinity (CrI) of the different samples was calculated using the following:

2.7 $$\mathrm{CrI}(\text{\%})=\frac{I_{002}-I_{\mathrm{am}}}{I_{002}}\times 100,$$

where *I*_002_ is the intensity of the 002 lattice plane at 2*θ* = 22.8°, and *I*_am_ is the intensity from the amorphous phase at approximately 2*θ* = 18° [15].

## 3. Results and discussion

### 3.1. Solubility of poplar wood meal in different DESs

**Figure 2.**
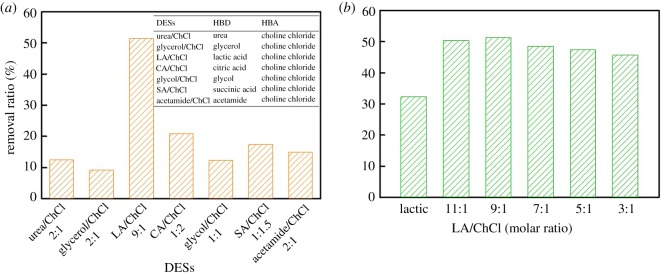
(*a*) Solubility of poplar wood meal in different DESs, and (*b*) the effect of the LA/ChCl molar ratio on the solubility of poplar wood meal.

Firstly, a series of DESs, composed of various hydrogen-bond donors and ChCl, were prepared to find the optimal DESs for the dissolution of poplar wood meal. Figure 2*a* presents the solubility of poplar wood meal in different DESs under 120°C for 6 h. As shown, the solubility of poplar wood meal in LA/ChCl is higher than that for other DESs, which is more than 50%, thereby suggesting that LA/ChCl is the optimal DES solvent for wood meal dissolution among these DESs. Furthermore, the effect of the LA/ChCl molar ratio on the solubility of poplar wood meal was investigated and is shown in figure 2*b*. The solubility of poplar wood meal increased with the LA/ChCl molar ratio (from 1 : 1 to 9 : 1), and LA/ChCl (9 : 1) exhibited the best dissolving capacity for poplar wood meal, which demonstrated LA plays a positive role in the solubility of polar wood meal. The chemical properties of DESs can be described by hydrogen-bond acidity, hydrogen-bond basicity and dipolarity/polarizability of DES solvent. The high hydrogen-bond acidity is conductive to breaking the lignin–carbohydrate complexes (LCCs) structure of wood meal, which is in agreement with Liu *et al.* [16]. The LA as a HBD mainly affects the hydrogen-bond acidity of DES solvent. The molar ratio of LA/ChCl (9 : 1) had the high hydrogen-bond acidity, enhancing the accessibility of wood meal and inducing disintegration. Furthermore, the utilization of DES can be described by Hole theory [17], which is related to the free volume and probability of finding holes for solvent molecules/ions to move into. Thus, a molar ratio of 9 : 1 was verified to be a priority in all ratios of LA/ChCl, but not 11 : 1. No waste was produced and the atom economy was nearly 100%. Thus, LA/ChCl (9 : 1) was chosen as the solvent system for wood meal and was further studied in the subsequent section.

**Figure 3.**
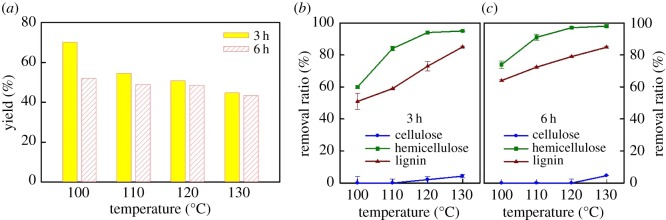
(*a*) Yield of solid residue (DES-C) after the LA/ChCl dissolving treatment, and the removal ratio of each component from poplar wood meal after treatment of LA/ChCl under different temperatures for (*b*) 3 h and for (*c*) 6 h.

The effects of the dissolving temperature and time with LA/ChCl on the solubility of polar powder are shown in figure 3. The yields of the solid residue (cellulose) after the LA/ChCl dissolving treatment decreased gradually with the increase of the temperature (figure 3*a*). Under the same LA/ChCl molar ratio, increasing temperature can maximize the ionic characteristics and increase the molecular polarity of the DES, promoting the breakage of the intramolecular hydrogen-bond network and enhancing the solubility of lignin and hemicellulose [6]. Under the same temperature, the solubility of LA/ChCl for poplar wood meal also showed an upward trend with the increase of the dissolving time. The solubility of LA/ChCl for poplar wood meal reached more than 50% at 130°C for 6 h.

The dissolving efficiency of LA/ChCl for each component in the raw material is calculated based on the contents of cellulose, lignin and hemicellulose in the solid residue (DES-C). As shown in figure 3*b,c*, LA/ChCl possesses better dissolving efficiency for lignin and hemicellulose than cellulose. The dissolving removal rates of lignin and hemicellulose can be improved with the increase of the temperature and time, both of which can reach more than 95%. Furthermore, the LA/ChCl treatment hardly dissolve cellulose when the treatment temperature is less than 130°C. Only a small amount of cellulose is dissolved in LA/ChCl when the temperature is up to 130°C; the cellulose content of 95% could still be retained in solid materials. The results indicated that LA/ChCl has a high affinity to lignin and hemicellulose. In addition, lignin is easy regenerated from hemicellulose by the addition of deionized water. Therefore, lignin with high purity could be acquired from poplar wood meal by the pretreatment of LA/ChCl. The results are similar to those of a previous report [5].

### 3.2. Recovery and purification of isolated lignin (DES-L)

**Figure 4.**
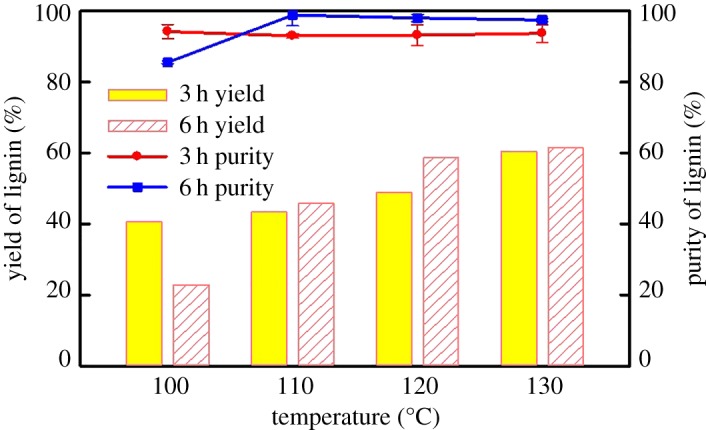
Yields and purity of isolated lignin (DES-L) separated by the LA/ChCl dissolving treatment under different dissolving temperatures and times.

After the LA/ChCl dissolving treatment was followed by separation by centrifugation, the solid residue was washed by acetone. The acetone soluble fraction was added into the supernatant, which contains dissolved hemicellulose and lignin. The isolated lignin can be precipitated by adding deionized water in this mixture solution after acetone removal while the hemicellulose remains in the solution (figure 1). The yields of DES-L and the lignin purities in DES-L under different dissolving temperatures and times are shown in figure 4. The yields of DES-L increase with the treatment temperature and time, and the trend is similar to the lignin solubility in LA/ChCl (figure 3*b*). The maximum recovered amount of DES-L is 61.5% from the LA/ChCl-treated poplar wood meal when the treatment temperature and time are 130°C and 6 h, respectively. Obviously, the yields of DES-L are less than the solubility of lignin with the LA/ChCl treatment (95%), which is attributed to that part of the lignin which is degraded to low-molecular-weight lignin and cannot be recovered. The purity analyses of DES-L under different temperatures and times are shown in figure 4. It can be seen from figure 4 that the purity of lignin increases with increase of reaction temperature from 100°C to 130°C which could ascribe to the more serious degradation of hemicellulose under higher temperature. In addition, the purity of lignin in regenerated products can reach more than 95% under the given condition, which suggests that DESs treatment provides a feasible method for the separation and extraction of lignin.

### 3.3. Structural characterization of isolated lignin (DES-L)

**Figure 5.**
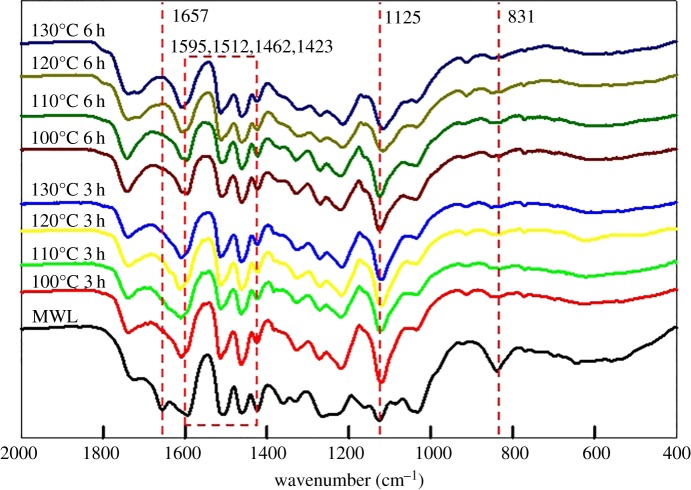
FT-IR spectra of the isolated lignin (DES-L) and milled wood lignin (MWL).

The structures of DES-L were characterized by FT-IR, nitrobenzene oxidation, quantitative ^13^C NMR, HSQC NMR and ^1^H NMR analyses. Milled wood lignin (MWL) made by the same poplar was used as a control sample to compare with the DES-L. Figure 5 shows the FT-IR spectra of the DES-L and MWL. The characteristic band for the aromatic ring vibrations are noticed at 1595, 1512, 1462 and 1423 cm^−1^ from lignin. The peaks at 1329/1125 cm^−1^, 1271 cm^−1^, 1224 cm^−1^ and 1036/831 cm^−1^ of lignin are assigned to the absorption vibrations of the clove nucleus, guaiacyl group, C–C/C–O/C=O vibrations of guaiacyl and C–H vibration, respectively [18]. Compared with MWL, a strong peak at 1740 cm^−1^ appeared and a peak from MWL at 1657 cm^−1^ disappeared, which originated from the non-conjugated carbonyl and ester group and the conjugated carbonyl vibrations. This finding suggests that the DES treatment may destroy the double bond of the lignin's alpha position and result in the destruction of the conjugated structure. Moreover, the treatment temperature and time only slightly affect the structure of the DES-L. Notably, the absence of a peak at 918 cm^−1^ demonstrates that there was no cellulose in regenerated products. The results indicated that high-purity lignin can be obtained by the DES treatment in this condition.

The nitrobenzene oxidation of raw material, solid residue and DES-L was performed to detect the effect of the DES dissolving treatment on the structure of lignin. The oxidation products are shown in table 1. The alkaline NBO products of poplar are vanillin (V), vanillic acid (VA), syringaldehyde (S), syringic acid (SA), 4-hydroxybenzaldehyde (H) and 4-hydroxybenzoic acid (HA). The main linkages of poplar lignin are non-condensable connections (α-O-4, β-O-4) and condensable connections (β-5, 5–5, β-1, 4-O-5). The alkaline NBO is used to investigate the condensable connections of lignin [19]. The yields of the S and V units are less than those of the H unit in DES-C, thereby implying that the DES solvent is ready to dissolve the S and V units. Compared with the NBO products of poplar wood meal and DES-L, the NBO products yields of DES-L have a significant reduction, which is attributed to the cleavage of a large amount of non-condensation bonds of lignin, such as α-aryl, alkyl and β-aryl ether bonds. The V units of DES-L decreased, because most of the α-O-4 bond is occupied by the guaiacyl unit, and the phenolic α-O-4 bond can be rapidly degraded at the initial delignification stage [20]. The value of M(S)/m(V) is 1.85, which belongs to the typical guaiacin syringin-based lignin.

**Table 1.** Yields and ratios of the nitrobenzene oxidation (NBO) products of lignin in poplar wood meal, solid residue (DES-C) and isolated lignin (DES-L).

samples	NBO products (mmol/g)				m(S)/m(V)	m(S)/m(V)/m(H)
	S	V	H	S + V+H		
polar powder	1.73	0.94	0.04	2.71	1.84	64 : 35 : 1
DES-C	0.31	0.65	1.75	2.71	0.48	15 : 32 : 87
DES-L	0.72	0.39	0.06	1.17	1.85	62 : 33 : 5

**Figure 6.**
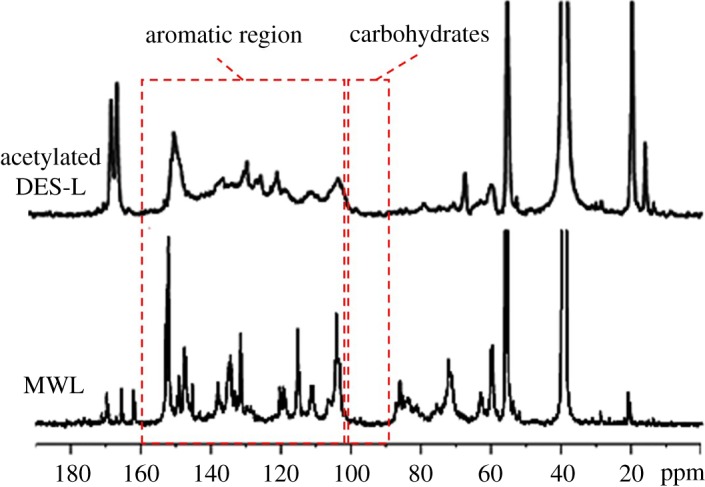
^13^C NMR spectra (ppm) of acetylated DES-L and MWL from poplar.

^13^C NMR spectroscopy is an informative tool for the determination of lignin and its derivative. This method provides not only the information of the phenylpropane units and the side chain linkages but also the different types of hydroxyl groups, including the primary, secondary, C_5_-free and C_5_-substituted hydroxyl groups, if the lignin is acetylated. Figure 6 presents the ^13^C NMR spectra of acetylated DES-L and MWL. The striking observation of the phenolic hydroxyl groups (174–170 ppm) and the weak signals of C_β_ in β-O-4 units (83.8 and 84.6 ppm) in DES-L confirm the cleavage of ether linkages, especially the β-aryl ether bonds [4]. The three broad categories of quaternary, oxygenated (C_Ar_-O), non-oxygenated (C_Ar_-C) and methine (C_Ar_-H), in the aromatic region of the lignin ^13^C NMR spectra (166–103 ppm), are important to quantify the different condensed moieties, such as the 5–5′ and 4-O-5′ linkages of phenolic and etherified types. Compared with MWL, the change of the DES-L signals in the aromatic region suggested that the side chains of lignin, especially the etherified structures, were damaged significantly after the DES treatment [21]. In addition, the absence of peak signals between 102 and 90 ppm, demonstrated that the carbohydrates were absent in DES-L. In other words, DES-L exhibited a high-purity property.

**Figure 7.**
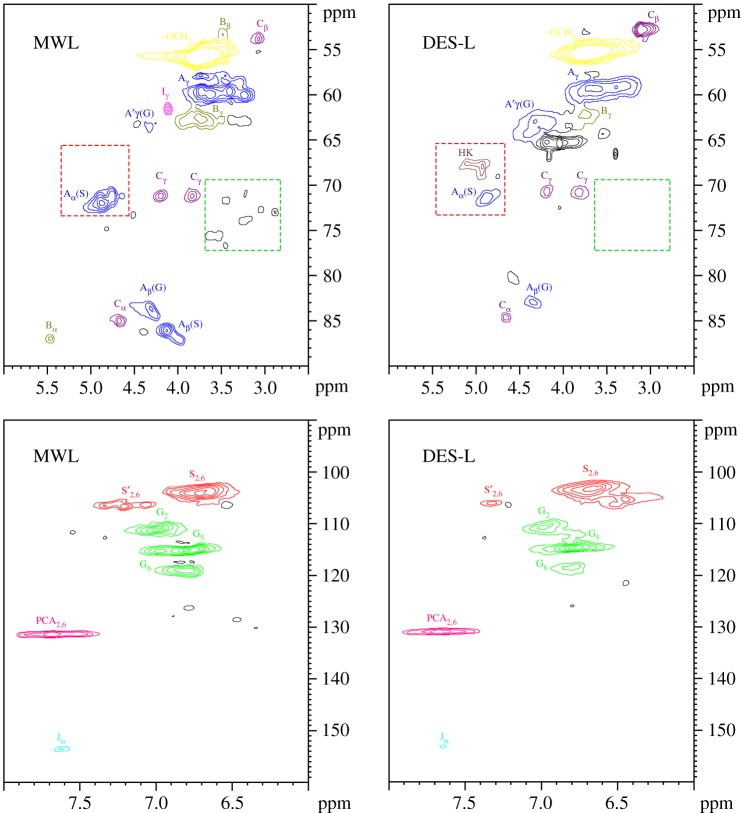
Side chain (*δ*_C_/*δ*_H_ 90–50/6.0–2.5) and aromatic (*δ*_C_/*δ*_H_ 160–90/8.0–6.0) regions of MWL and DES-L in the 2D HSQC NMR spectra.

**Figure 8.**
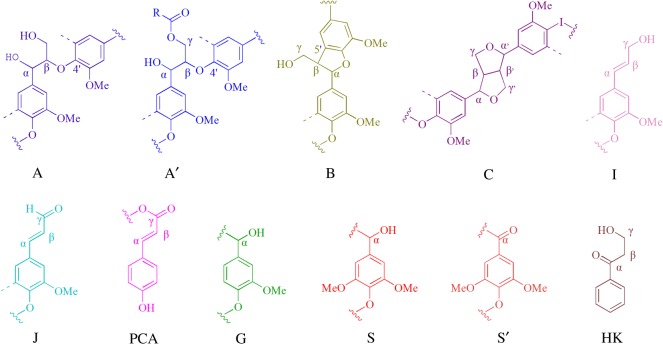
Main linkages of lignin units identified by 2D NMR. (A, A′) β-O-4 ether linkages, (B) phenylcoumarin, (C) resinol, (I) cinnamyl alcohol group, (J) cinnamaldehyde, (PCA) p-coumarate, (G) guaiacyl, (S, S') syringyl, and (HK) the unit after cleavage of β-O-4.

HSQC NMR is another effective tool to probe the structure of lignin. This method can determine the specific carbon–hydrogen functionalities that are unable to be identified in the ^13^C and ^1^H spectra. The side chain (*δ*_C_/*δ*_H_ 90–50/6.0–2.5) and aromatic (*δ*_C_/*δ*_H_ 160–90/8.0–6.0) regions of the HSQC NMR spectra of DES-L and MWL are shown in figure 7, and the structures of the main lignin substructures are depicted in figure 8. The correlation peaks from methoxyl and β-O-4′ substructures (A, A’) were the most prominent in the HSQC spectra of MWL. Nevertheless, that obviously changed after DES treatment, especially for the β-O-4′ substructures. The signals of C_α_-H*_α_* and C_β_-H_β_ in the A substructure became very weak, thereby indicating the oxidation of C*_α_* and the cleavage of β-O-4 linkages. However, the signal intensity of the C_γ_-H*_γ_* correlations in the A’ substructure of DES-L was considerably higher than that of MWL, which implied that esterification may occur between the γ-OH in the A substructure and LA. Additionally, the signals of the B substructure were scarcely observed in the spectrum of DES-L, but those of the C substructure were almost unchanged compared with the HSQC NMR spectrum of the MWL, thereby indicating the β-β, α-O-γ and γ-O-α linkages were more stable than the β-5 and α-O-4 bonds in DES. It is intriguing that Hibbert's ketone (HK) linkages were detected in DES-L. In consideration of the analysis of the β-O-4 linkages, it is reasonable to deduce that the HK structure is mainly from the degradation of the β-O-4 structure. In addition, the absence of the carbohydrate signal in the side chain regions of the DES-L suggested that the DES-L possesses high purity, which is in good agreement with the analysis of ^13^C NMR. The aromatic regions of MWL and DES-L in the HSQC NMR spectra were similar with the main signals corresponding to guaiacyl and sringyl units. This finding indicates that the fragmentization of lignin is mainly caused by the degradation of the side chains. The prominent signals assigned to p-coumarate were also observed, but the ferulate structure was only slightly detected in MWL and DES-L. During lignification, the p-coumarate and ferulate are frequently reduced to corresponding aldehydes, and these reactions are catalyzed by cinnamate: CoA ligase, which is distributed in various higher plants [22]. However, ferulate just provides the initiation sites from which the lignification event in the cell wall begins and p-coumarate is present via lignification using monolignol conjugation [23]. Therefore, the p-coumarate structure was easily detected, but the ferulate structure was hardly detected in the 2D HSQC spectra.

Besides, as shown in figure 3, almost 95% of cellulose remained in the residue under all the examined conditions, suggesting that DESs are good solvents for the separation of cellulose from poplar wood meal. The property of solid residue (DES-C) from poplar wood meal was further characterized by XRD (electronic supplementary material, figure S1) and FT-IR (electronic supplementary material, figure S2), to verify the effects of DES pretreatment on cellulose. The DES-C had the typical cellulose I structure with characteristic peaks at 2*θ* = 16.3° and 22.5° that were assigned to diffraction planes of 110 and 200 [24], respectively. The cellulose crystallinities of treated DES-C are 72.1, 68.0, 67.2, 71.2 and 64.7 for the treatment temperature/time of 120°C/3 h, 130°C/3 h, 110°C/6 h, 120°C/6 h and 130°C/6 h, respectively. The small difference in cellulose crystallinities suggested that the DES dissolving treatment hardly affected the crystal structure of cellulose under the studied dissolving temperature and time. From the FT-IR spectra, the presence of the adsorption peak of 1738 cm^−1^ is because the esterification of cellulose and LA at a high temperature created numerous C = O bonds. The adsorption peak at 1633 cm^−1^ is ascribed to the hydrogen bond formed by the hydroxyl groups in cellulose. In addition, the peak intensity at 1738 cm^−1^ increased, and the peak intensity at 1633 cm^−1^ decreased with the increase in the dissolving time, which further confirmed the esterification of cellulose and LA during treatment. The absence of adsorption peaks at 1595, 1512, 1462 and 1423 cm^−1^ indicated no benzene ring in DES-C, which suggested that a large amount of lignin can be removed from raw materials through the DES-dissolving treatment.

## 4. Conclusion

In this work, a feasible method of using DESs for lignin selective isolation from wood meal was provided. This method exhibited the following features: (i) the DES LA/ChCl has an excellent selective dissolving property for lignin. The optimal dissolving capacity can reach 95% when the molar ratio of LA/ChCl is 9 : 1 at 120°C for 6 h; (ii) the purity of regenerated lignin (DES-L) is up to 98.1%, and the FT-IR spectra demonstrated no cellulose in DES-L; (iii) the structural characterization of DES-L, such as nitrobenzene oxidation and NMR analysis, suggested that the DES can selectively cleave ether linkages and damage the non-condensation sections of lignin, thereby facilitating lignin dissolution from wood; and (iv) the content of cellulose in solid residue (DES-C) is more than 90%, and the crystal structure of cellulose changed only slightly as observed by XRD, thereby indicating that DES is a promising solvent for the separation of wood components.

## Supplementary Material

Supplementary Figures

## Data accessibility

The datasets supporting this article have been uploaded as part of the electronic supplementary material: https://dx.doi.org/10.6084/m9.figshare.7229222.
