# Supplementary material for: High-purity lignin isolated from poplar wood meal through dissolving treatment with deep eutectic solvents
Source: R Soc Open Sci. 2019 Jan 23;6(1):181757. doi: 10.1098/rsos.181757 (PMC6366171; doi:10.1098/rsos.181757)
Supplement: Supplementary Figures [file rsos181757supp1.docx]

*Electric supplementary material*

High-purity lignin isolated from poplar wood meal through dissolving treatment with deep eutectic solvents

Yujie Chen, Lili Zhang, Juan Yu, Yingzhao Lu, Bo Jiang, Yimin Fan, Zhiguo Wang †

Jiangsu Co-Innovation Center of Efficient Processing and Utilization of Forest Resources, Nanjing Forestry University, Nanjing 210037, China.

**Keywords:** deep eutectic solvents; lignin isolation; selective extraction; high purity

Figure s1. X-ray diffraction pattern of residue at different treatment temperatures and times.

Figure s2. FT-IR spectra of residue at different treatment temperatures and times.
